# Prognostic potential of the *MDM2* 309T>G polymorphism in stage I lung adenocarcinoma

**DOI:** 10.1002/cam4.750

**Published:** 2016-05-26

**Authors:** Yasuaki Enokida, Kimihiro Shimizu, Jun Atsumi, Seiichi Kakegawa, Yoshiaki Takase, Kyoichi Kaira, Hideaki Yashima, Takuya Araki, Seshiru Nakazawa, Yoichi Ohtaki, Toshiteru Nagashima, Lezhava Alexander, Kengo Usui, Toshihisa Ishikawa, Yoshihide Hayashizaki, Izumi Takeyoshi

**Affiliations:** ^1^ Department of Thoracic and Visceral Organ SurgeryGunma University Graduate School of MedicineMaebashiGunma371‐8511Japan; ^2^ Department of Oncology Clinical DevelopmentGunma University Graduate School of MedicineMaebashiGunma371‐8511Japan; ^3^ Department of Clinical PharmacologyGunma University Graduate School of MedicineMaebashiGunma371‐8511Japan; ^4^ Division of Genomic TechnologiesRIKEN Center for Life Science TechnologiesYokohamaKanagawa230‐0045Japan; ^5^ NGO Personalized Medicine & HealthcareYokohamaKanagawa226‐0016Japan; ^6^ RIKEN Preventive Medicine and Diagnosis Innovation ProgramYokohamaKanagawa230‐0045Japan

**Keywords:** Lung cancer, *MDM2*, *p53*, polymorphism, prognosis, SNP, SNP309

## Abstract

The MDM2 protein plays an important role in the regulation of cell proliferation and apoptosis via ubiquitination and proteasome‐mediated degradation of p53. The genetic polymorphism rs2279744 (c.309T>G) of the *MDM2* gene is reportedly associated with susceptibility and/or prognosis in various cancers. In this study, we investigated the risk factors for worse survival in patients with lung adenocarcinoma (AC). We examined the association between c.309T>G and the prognosis of lung cancer by retrospectively reviewing 453 lung cancer patients. We studied both, clinicopathological and genetic characteristics, including the c.309T>G, *p53* Arg72Pro, *EGFR*,*KRAS*, and *p53* mutations. Associations between these factors and survival outcome were analyzed using Cox proportional hazards models. The frequencies of *MDM2* polymorphisms were T/T, 20.8%; T/G, 48.6%, and G/G, 30.7%. The overall survival (OS) of AC patients with pathological stage I disease and the *MDM2* T/T genotype was significantly shorter than that of those with the T/G or G/G genotypes (*P* = 0.02). Multivariate analysis revealed that the *MDM2* T/T genotype was an independent, significant prognostic factor (hazard ratio [HR] = 2.23; 95% confidence interval [CI]: 1.07–4.65; *P *=* *0.03). The *MDM2* T/T genotype was predictive of poorer survival in a Japanese population. Genotyping for this polymorphism might predict the clinical outcomes of stage I AC patients.

## Introduction

Lung cancer is the leading cause of cancer‐related mortality worldwide 1. Although the overall incidence of lung cancer has been declining, particularly in Western countries, an increase in the proportion of lung adenocarcinoma (AC) is evident 2. The 5‐year survival rates for completely resected lung cancer were 86.8% (pathological [p‐] stage IA) and 73.9% (p‐stage IB) in Japan 3. A certain number of p‐stage I lung cancers paradoxically relapse even after surgical resection of the primary lesion and histopathological absence of any lymph node metastasis. This indicates that a small proportion of early‐stage lung cancers have highly metastatic characteristics. Therefore, screening possible high‐risk patients for disease recurrence is necessary to provide tailored medicine.

The *p53* gene is a well‐known tumor suppressor that is frequently mutated in non‐small‐cell lung cancer (NSCLC) patients 4. *p53* encodes a sequence‐specific DNA‐binding transcription factor targeting various genes that govern specific cellular processes 5. The MDM2 protein plays an important role in regulating cell proliferation and apoptosis by mediating ubiquitination and proteasome‐mediated degradation of p53 after binding directly to the latter protein; MDM2 has an E3 ubiquitin ligase activity 6, 7. A single‐nucleotide polymorphism (SNP) in the *MDM2* promoter region, a T‐to‐G change at nucleotide c.309 (rs2279744) in the first intron (c.309T>G), increases the binding affinity toward stimulatory protein 1 (Sp1), causing higher‐level MDM2 expression 8. Also, cells harboring homozygous 309G alleles express higher levels of MDM2 protein, thereby reducing the tumor‐suppressing activity of p53 8. In humans, c.309T>G is associated with earlier onset of tumor formation in both hereditary and sporadic cancers 9. Recently, another antagonizing MDM2 polymorphism, SNP285, has been reported^10^ among Caucasians. SNP285 has been reported to nullify the effect of SNP309 and to reduced risk of breast, endometrial, and ovarian cancer. Molecular epidemiological studies of the c.309T>G polymorphisms in terms of lung cancer susceptibility^11^, ^12^, ^13^ have yielded contradictory findings. We recently reported that c.309T>G was not associated with lung cancer susceptibility in a Japanese population 14. The effects of c.309T>G on lung cancer survival have reported first in 2007^15^ and remain controversial 12, 15, 16, 17, 18, 19, 20. So far, seven studies have analyzed the association between c.309T>G of the *MDM2* gene and lung cancer prognosis. The G allele was reported to be a poor prognosis factor in Caucasians and Asians 15, 16. However, recently, some reports^17^, ^20^concluded that the T allele was a poor prognosis factor in Asians. Furthermore, three reports found no association between SNP309 and lung cancer survival in Asian 18, 19, Caucasian and African‐American 12.

In this study, we investigated whether c.309T>G of the *MDM2* gene is closely associated with survival outcome of surgically resected NSCLC together with other clinicopathological and genetic characteristics.

## Patients and Methods

### Study population

To carry out this clinical research, we obtained approval from the Institutional Review Board of the Ethical Committee for Human Genome Analysis at Gunma University, and written informed consent from all the patients who participated. We analyzed 453 consecutive lung cancer patients (stages I–III) surgically treated between January 2003 and December 2012 at the Department of Thoracic and Visceral Organ Surgery, Gunma University Graduate School of Medicine, Gunma, Japan. Patients who had undergone preoperative therapies (chemotherapy and/or radiation therapy) and had a history of lung cancer were excluded. History of cancer and smoking were documented using a chart review before surgery. Never smokers were defined as individuals with a lifetime exposure to fewer than 100 cigarettes. Other patients were defined as smokers these include both former and current smoker. Disease staging was used to divide the patients into two groups: those of stages I and II–III. All the pathological factors, including pleural, vascular, and lymphatic invasion, were documented from the pathologic analysis at Gunma University Hospital. Cases that were positive for vascular invasion or lymphatic invasion were defined as lymphovascular invasion (LVI) positive. All the patients were reclassified according to the 7th edition of the International Union against Cancer (UICC) tumor‐node‐metastasis (TNM) staging system 21. The type of treatment after cancer recurrence was chosen by each individual physician. Overall survival (OS) was determined as the time from tumor resection to death from any cause. Disease‐free survival (DFS) was defined as the time between tumor resection and the first disease progression or death. All research followed the principles of the Declaration of Helsinki.

### SNP genotyping

Peripheral venous blood samples were collected, and DNA was extracted using the DNeasy Blood & Tissue Kit (Qiagen, Hilden, Germany) according to the manufacturer's instructions. The DNA was used for SNP typing of c.309T>G and *p53* Arg72Pro polymorphisms. Genotyping of c.309T>G was carried out using the Duplex SmartAmp method as described previously 22. *p53* Arg72Pro was genotyped using polymerase chain reaction‐restriction fragment length polymorphism (PCR‐RFLP) based on a previous report 23. Subsequently, the samples representing each genotypic pattern were used as controls in each assay.

### Gene mutation analysis

Tissue samples from patients were isolated from surgically resected lung tumors. Lung cancer tissues were immediately frozen after surgical removal and stored at −80°C until DNA extraction using the Wizard Genomic DNA Purification Kit (Promega, Madison, WI). The genomic DNA was used as a template for mutation analysis of *EGFR*,* KRAS*, and *p53*. *KRAS* and *EGFR* mutations were analyzed by sequencing as described previously 24, 25. Mutations in exons 5–8 of *p53* were detected by direct sequencing 26. Briefly, primers used in the reactions were E5‐6S (5′‐TGCCCTGACTTTCAACTCTG‐3′) and E5‐6AS (5′‐AGTTGCAAACCAGACCTCAGG‐3′) for exons 5 and 6, and E7‐8S (5′‐CTTGCCACAGGTCTCCCCAA‐3′) and E7‐8AS (5′‐TCTCCTCCACCGCTTCTTGT‐3′) for exons 7 and 8. All the *p53* mutations were confirmed by sequencing of both DNA strands.

### Statistical analyses

Probability values less than 0.05 indicated a statistically significant difference. Differences in the distributions between groups were examined by Pearson *χ* ^2^ tests. Kaplan–Meier curve and the log‐rank test were used to estimate differences in survival. Hazard ratios (HRs) from univariate Cox regression analysis were used to determine the association between clinic‐pathological features and OS. Variables with statistically significant differences in univariate analysis were entered into multivariate analysis. Multivariate Cox proportional hazards regression was used to evaluate independent prognostic factors. All the statistical analyses were performed using SPSS Statistics version 20 (IBM Co., NY, USA).

## Results

### Demographics of patients according to *MDM2* genotype

Table 1 depicts the characteristics of the entire study population. The study population was composed of 260 males and 193 females of a median age 68 years (range, 33–87 years). The genotype frequencies of *MDM2* polymorphisms were as follows: T/T, 20.8%; T/G, 48.6%; and G/G, 30.7%. The frequency of the *MDM2* 309G allele was 0.55, consistent with previously described values for Asian lung AC patients 22.

**Table 1:** Patients characteristics

	*n*	%
Sex		
Women	193	42.6
Men	260	57.4
Age		
Mean ± SD	68.1 ± 9.5	
Smoking status		
Never smoker	179	59.5
Smoker	274	60.5
Surgical procedure		
≥Lobectomy	388	85.7
≤Segmentectomy	65	14.3
Pathological stage		
I	322	71.1
II	60	13.2
III	71	15.7
T factor		
T1	218	48.1
T2	194	42.8
T3	39	8.6
T4	2	0.4
N factor		
N0	348	76.8
N1	42	9.3
N2	63	13.9
Histology		
AC	328	72.4
SQ	107	23.6
Others	18	4.0
MDM2 SNP309		
TT	94	20.8
TG	220	48.6
GG	139	30.7
Adjuvant chemotherapy	123	27.2
Chemotherapy (postrecurrence)	69	15.2
Radiation therapy (postrecurrence)	51	11.3

Patients who had undergone preoperative therapies (chemotherapy and/or radiation therapy) and had a history of lung cancer were excluded. AC, adenocarcinoma; SQ, squamous cell carcinoma.

### Survival analysis

The median follow‐up time was 56.5 months (range, 1.1–150 months). The 5‐year OS and DFS rates of the total study population were 73.7% (95% confidence interval [CI]: 69.2–78.2) and 66.1% (95% CI: 61.4–20.8), respectively. The percentages of patients treated via chemotherapy and/or radiotherapy after recurrence did not significantly differ (*P* = 0.156; Pearson *χ* ^2^ test). Table 2 shows the results of HR adjusted for age, sex, stage, histology, treatment (chemotherapy after tumor recurrence), and smoking status. Although no association was observed between this polymorphism and the population as a whole, the *MDM2* T/T genotype was significantly associated worse DFS and OS among AC patients and p‐stage I AC patients.

**Table 2:** Hazard ratios for survival data according to *MDM2* genotypes. T/G + G/G (Reference: HR = 1.0) vs. T/T

				DFS				OS			
Histology	Pathological stage		*N*	Event	HR	95% CI	*P*	Event	HR	95% CI	*P*
All	Stage I‐III	a	453	153	1.15	078–1.69	0.48	118	1.01	0.64–1.58	0981
AC		b	328	99	1.93	1.21–3.07	**0.01**	73	2.05	1.19–3.51	**0.01**
SQ		b	107	54	0.71	0.32–1.53	0.38	45	0.50	0.20–1.28	0.15
AC	Stage I	b	246	47	2.11	1.13–3.95	**0.02**	35	3.00	1.44–6.24	**0.003**
	Stage II, III	b	82	52	1.78	0.87–3.67	0.12	38	1.33	0.54–3.30	0.54
SQ	Stage I	b	63	24	0.75	0.21–2.67	0.66	18	0.34	0.04–2.67	0.30
	Stage II, III	b	44	30	0.65	0.22–1.92	0.44	27	0.65	0.19–2.17	0.48

*P* < 0.05 are shown in bold. DFS, disease‐free survival; OS, overall survival; AC, adenocarcinoma; SQ, squamous cell carcinoma.

a Hazard ratio adjusted for age, sex, stage, histology, treatment (chemotherapy after tumor recurrence), and smoking status.

b Hazard ratio adjusted for age, sex, treatment (chemotherapy after tumor recurrence), and smoking status.

### Subgroup analysis of stage I AC patients

**Figure 1:**
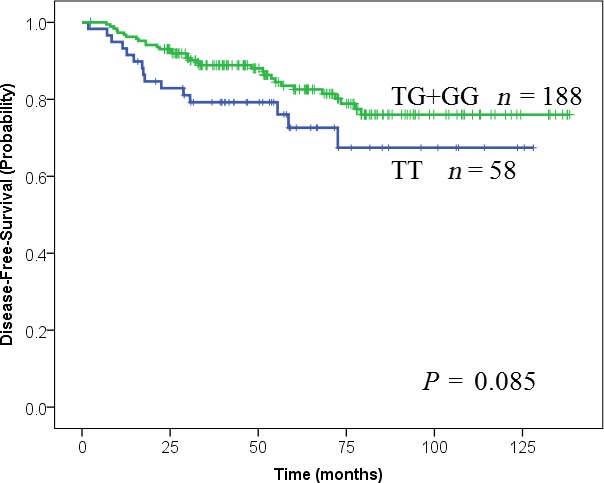
Kaplan–Meier estimates of disease‐free survival in patients with stage I lung adenocarcinoma. *MDM2* c.309T>G (T/T, blue; T/G + G/G, green). The *P* value was calculated using the log‐rank test.

**Figure 2:**
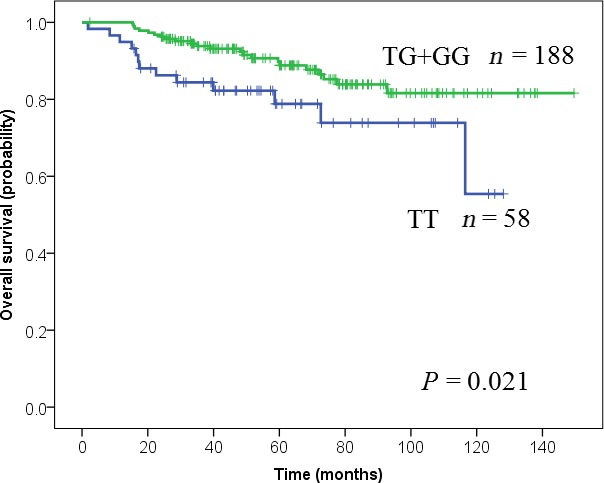
Kaplan–Meier estimates of overall survival in patients with stage I lung adenocarcinoma. *MDM2* c.309T>G (T/T, blue; T/G + G/G, green). The *P* value was calculated using the log‐rank test.

Among stage I AC patients, a significant association was found between smoking status, pleural invasion, or LVI and c.309T>G (T/T vs. T/G + G/G).(Table 3). Figures 1 and 2. show Kaplan–Meier survival curves of AC p‐stage I patients according to *MDM2* genotype for DFS and OS, respectively. The OS of patients with the T/T genotype was shorter than the OSs of patients with the G/G or T/G genotypes (*P* = 0.021; log‐rank test). The 5‐year OS and DFS rates of the total population were 86.4% (95% CI: 81.7–91.1) and 80.2% (95% CI: 74.5–85.9), respectively. Together with Kaplan–Meier analysis, we compared the T/T genotype with G allele carriers (T/G + G/G) using univariate and multivariate analyses. The results of univariate analysis for OS are summarized in Table 4. The HR for death in the T/T group relative to the T/G + G/G group was 2.20 (95% CI: 1.10–4.36; *P* = 0.025). Similarly, the clinicopathological factors (gender, age, smoking history, differentiation, LVI, pleural invasion, and *EGFR* mutation) significantly affected survival. Conversely, the status of the *KRAS* and *p53* mutations and p53codon72 were not significant upon univariate analysis (Table 4). Multivariate analyses for OS revealed that MDM2 T/T genotype was a significant independent risk factor (HR = 2.23; 95% CI: 1.07–4.65; *P* = 0.033), together with male gender (HR = 5.69; 95% CI: 1.78–18.2; *P* = 0.003), older age (HR = 2.39; 95% CI: 1.12–5.09; *P* = 0.002) and LVI (HR = 1.58; 95% CI: 1.01–2.47; *P* = 0.044) (Table 4).

**Table 3:** Patient characteristics of stage I AC patients according to *MDM2* genotypes

		*MDM2* genotypes						T/T vs. T/G vs. G/G	T/T vs. T/G + G/G
	Total	T/T		T/G		G/G		*P* a	*P* a
All cases	246	58	23.6	115	46.7	73	29.7	–	–
Sex									
Women	134	27	20.3	65	48.9	41	20.8	0.42	0.23
Men	113	31	27.4	50	44.2	32	28.3		
Age in years									
<70	135	33	24.4	63	46.7	39	28.9	0.92	0.76
≥70	111	25	23.6	52	46.8	34	30.6		
Smoking status									
Never smoker	140	24	17.1	73	52.1	43	30.7	**0.02**	**0.01**
Smoker	106	34	32.1	42	39.6	30	28.3		
*p53* Arg72Pro									
Arg/Arg	90	20	22.2	42	46.7	28	31.1	0.90	0.93
Arg/Pro	115	28	24.3	56	48.7	31	27.0		
Pro/Pro	41	10	24.4	17	41.5	14	34.1		
Performance status^b^									
0	208	49	23.6	101	48.6	58	27.9	0.30	1.00
1–2	21	5	23.8	7	33.3	9	42.9		
Surgical procedure									
Lobectomy	201	49	24.4	91	45.3	61	30.3	0.61	0.70
Segmentectomy	45	9	20.0	24	53.3	12	26.7		
Differentiation									
Well	137	30	21.9	69	50.4	38	27.7	0.44	0.55
Moderate or poorly	109	28	25.7	46	42.2	35	32.1		
T factor									
T1	158	35	21.5	71	44.9	53	33.5	0.19	0.35
T2	88	24	27.3	44	50.0	20	22.7		
Pleural invasion									
Absent	202	42	20.8	96	47.5	64	31.7	0.07	**0.03**
Present	44	16	36.4	19	43.2	9	20.5		
Lymphovascular invasion									
Absent	177	34	19.2	92	52.0	51	28.8	**0.01**	**0.01**
Present	69	24	34.8	23	33.3	22	31.9		
*p53* status									
Wild type	207	48	23.2	98	47.3	61	29.5	0.90	0.83
Mutant	39	10	25.6	17	43.6	12	30.8		
*EGFR* status^b^									
Wild type	133	30	22.6	62	46.6	41	30.8	0.92	0.76
Mutant	111	27	24.3	52	46.8	32	28.8		
*KRAS* status									
Wild type	214	54	25.2	99	46.3	61	28.5	0.25	0.13
Mutant	32	4	12.5	16	50.0	12	37.5		
Adjuvant chemotherapy									
Received	47	14	29.8	27	57.4	6	12.8	0.02	0.26
Not received	199	44	22.1	88	44.2	67	33.7		
Chemotherapy									
Received	16	3	18.8	9	56.2	4	5.5	0.80	0.55
Not received	11	4	36.4	4	36.4	3	27.31		

NA, not available; AC, adenocarcinoma.

a *P* values were calculated by chi‐square test. *P *< 0.05 are shown in bold.

b Performance status at surgery and EGFR mutation status remains unknown in some cases.

**Table 4:** Univariate and multivariate analysis in disease‐free survival and overall survival of stage I AC patients

	DFS						OS					
	Univariate analysis			Multivariate analysis			Univariate analysis			Multivariate analysis		
Variables	HR	95% CI	*P* a	HR	95% CI	*P* a	HR	95% CI	*P* a	HR	95% CI	*P* a
Sex												
Women	1.0			1.0			1.0			1.0		
Men	3.32	1.77–6.21	**<0.0001**	2.11	0.90–4.98	0.087	7.39	3.05–17.9	**<0.0001**	5.69	1.78–18.2	**0.003**
Age												
<70	1.0			1.0			1.0			1.0		
≥70	2.75	1.50–5.03	**0.001**	1.95	1.04–3.66	**0.039**	3.07	1.50–6.27	**0.002**	2.39	1.12–5.09	**0.024**
Smoking history												
Never smoker	1.0			1.0			1.0			1.0		
Smoker	3.4	1.84–6.29	**<0.0001**	0.919	0.60–1.42	0.22	4.73	2.21–10.1	**<0.0001**	1.14	0.69–1.91	0.089
*p53* Arg72Pro												
Arg/Arg + Arg/Pro	1.0						1.0					
Pro/Pro	0.82	0.37–1.83	0.62				0.80	0.31–2.07	0.65			
*MDM2* c.309T>G												
T/G + G/G	1.0			1.0			1.0			1.0		
T/T	1.71	0.92–3.15	**0.09**	1.45	0.76–2.76	0.254	2.20	1.10–4.36	**0.025**	2.23	1.07–4.65	**0.033**
Performance status												
0	1.0						1.0					
1,2	0.94	0.29–3.05	0.92				1.34	0.41–4.43	0.63			
Differentiation												
Well	1.0			1.0			1.0			1.0		
Moderate and poorly	3.94	2.08–7.48	**<0.0001**	0.79	0.54–1.16	0.22	4.76	2.16–10.5	**<0.0001**	0.79	0.49–3.31	0.34
T factor												
T1	1.0						1.0			1.0		
T2	2.93	1.64–5.23	**<0.0001**	1.76	0.84–3.72	0.14	2.62	1.34–5.11	**0.005**	1.27	0.49–3.31	0.62
Lymphovascular invasion												
Negative	1.0						1.0			1.0		
Positive	4.36	2.43–7.82	**<0.0001**	1.48	1.04–2.10	**0.03**	6.10	3.00–12.4	**<0.0001**	2.50	1.03–6.11	**0.044**
Pleural invasion												
Negative	1.0			1.0			1.0			1.0		
Positive	2.49	1.35–4.60	**0.004**	1.19	0.542–2.61	0.66	3.00	1.51–5.96	**0.002**	1.13	0.44–2.90	0.80
*EGFR* gene mutation												
Mutant	1.0						1.0			1.0		
Wild type	2.02	1.08–3.78	0.027				2.94	1.34–6.48	**0.007**	1.46	0.60–3.56	0.41
*KRAS* gene mutation												
Wild type	1.0						1.0					
Mutant	1.13	0.51–2.53	0.76				1.31	0.54–3.16	0.55			
*p53* gene mutation												
Wild type	1.0						1.0					
Mutant	1.43	0.69–2.97	0.33				1.75	0.79–3.84	0.17			
Adjuvant chemotherapy												
Not received	1.0						1.0					
Received	1.56	0.79–3.07	0.2				1.25	0.54–2.89	0.6			

HR and 95% CI are shown as the values of the latter compared to the former (HR = 1.0). *P *< 0.05 are shown in bold. DFS, disease‐free survival; OS, overall survival; HR, hazard ratio; CI, confidence interval.

a *P* values were calculated by Cox regression analysis.

### Stratified analyses of the prognostic effects of the *MDM2* genotypes

**Figure 3:**
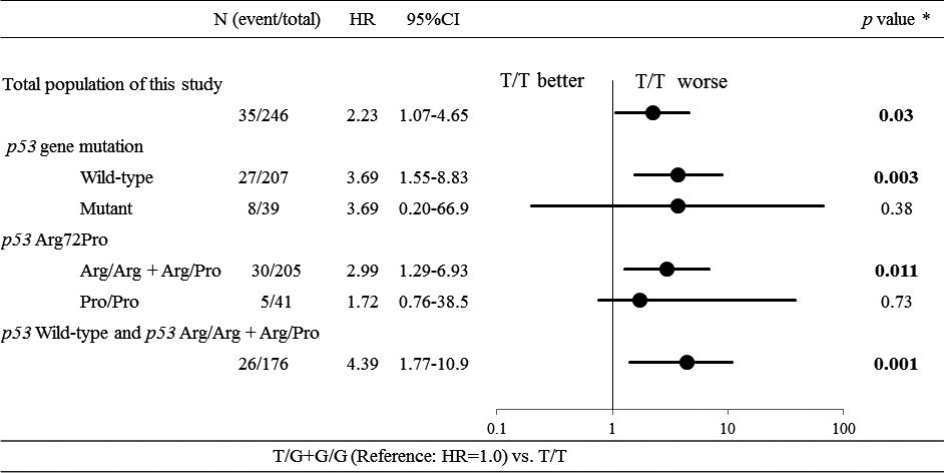
Subset analysis of overall survival in stage I lung adenocarcinoma. The forest plot shows the multivariate Cox regression for each subgroup. *P* values correspond to hazard ratios adjusted by multivariate regression. The *P* values <0.05 are shown in bold.

We further evaluated the associations between the prognostic effects of c.309T>G of the *MDM2* gene and *p53* status using stratified analyses (Fig. 3). Stronger relationships were observed among *p53* wild‐type group (HR = 3.69) and *p53* Arg72Pro Arg/Arg + Arg/Pro group (HR = 2.99). Further stratified analyses of patients with a *p53* wild‐type tumor and Arg/Arg + Arg/Pro genotype of *p53* Arg72Pro showed a higher (HR = 4.39), but these results are underpowered due to small sample size.

## Discussion

Because disruption of *p53* tumor suppressor function is important in cancer development, we hypothesized that the 309G allele of the *MDM2* gene would be associated with worse survival outcomes among surgically treated lung cancer patients. Unexpectedly, our study demonstrated that the T/T genotype of c.309T>G was a significant independent unfavorable prognostic factor, and the associated tumors tended to show pleural invasion or LVI among stage I lung AC patients.

So far, seven studies have analyzed the association between c.309T>G of the *MDM2* gene and lung cancer prognosis, but the results were contradictory (Table 5). Our present results are consistent with the two reports from Taiwan and China 17, 20. Heist et al. 15. investigated the impact of *MDM2* gene polymorphism in early‐stage (stage I or II) NSCLC patients in the United States and reported that the G/G genotype was associated with worse OS. These findings might seem contrary to our results. However, when analyzed in detail, Heist's subgroup analyses (Table 5) and ours had similar results. First, Heist showed that the G/G genotype was associated with worse OS only in patients with stage IB/II NSCLC and squamous cell carcinoma (SQ) histology 15. Similarly, we showed that the T/T genotype was associated with better OS in patients with SQ histology (although it did not reach statistical significance). Next, Heist showed that, the G/G genotype was associated with better OS in patients with stage IA NSCLC or those with AC histology (although not significant) 15. This is consistent with our results (Table 2), since we showed that the T/T genotype was associated with worse OS in patients with stage I NSCLC or those with AC histology. We believe that the difference in statistical power between the two studies may be due to the difference of study population. Recently, SNP285 has been reported to act as an antagonist to SNP309 only observed in Caucasians 10, furthermore, Ryan et al. 12. showed that neither SNP309 nor SNP285 were associated with lung survival. Therefore, this point is still a matter of debate. SNP285 per se could not explain the discrepancies between Heist's study and ours. Han et al.^[^ 16 and Liu et al. 19. investigated stage III or IV NSCLC patients and reported disparate findings. Survival outcome of advanced lung cancer depends strongly on tumor size, lymph node metastasis, or therapeutic regimen. Therefore, known genetic factors might have less influence on cancer prognoses if study subjects have only advanced‐stage NSCLC. Our results support the previous study from Asia (Taiwan) focusing on stage I NSCLC^17^ and which reported a tendency for the T/T group to be a poor prognostic factor compared to the G/G group (*P *=* *0.05).

**Table 5:** Comparison of previous reports concerning c.309T>G and lung cancer prognosis

Author	Year	Country	Ethnic group	Smoker (%)	Stage	Histology	Subgroup	N	*MDM2* c.309T>G SNP309	HR	95% CI	*P*	Correlation	Ref.
Heist	2007	States	Caucasian	93%	Stage I, II	NSCLC		383	T//T vs. G/G	1.57	1.03–2.40	0.04	G/G risk	15
							AC	186	T/T vs. G/G	0.93	0.48–1.80	0.82	NS	
							SQ	110	T/T vs. G/G	3.77	1.88–7.57	0.0002	G/G risk	
							Stage IA	200	T/T vs. G/G	0.65	0.33–1.27	0.2	NS	
							Stage IB or II	183	T/T vs. G/G	3.19	1.80–5.65	<0.0001	G/G risk	
							Noncurrent smoker	233	T/T vs. G/G	1.17	0.66–2.08	0.58	NS	
							Current smoker	150	T/T vs. G/G	2.93	1.52–5.67	0.001	G/G risk	
Han	2008	Korea	Asian	75%	Stage IIIB or IV	NSCLC		148	T/T vs. T/G + G/G	1.74	1.05–2.89	0.032	T/G + G/G risk	16
Chua	2010	Singapore	Asian	0%		Lung cancer		123	T/T vs. T/G vs. G/G	NA		0.27(log‐rank)	N.S.	18
Chien	2010	Taiwan	Asian	51%	Stage I‐III	NSCLC		198	T/T vs. G/G	0.62	0.41–0.95	0.003	T/T risk	17
							Stage I	127	T/T vs. G/G	0.47	0.22–1.01	0.05	NS	
							Stage I with wild‐type *p53*	99	T/T vs. G/G	0.34	0.15–0.80	0.01	T/T risk	
							Stage I with *p53* mutation	28	T/T vs. G/G	5.02	0.31–28.20	0.35	NS	
							Stage II, III	179	T/T vs. G/G	0.82	0.48–1.39	0.45	NS	
Liu	2011	China	Asian	59%	Stage III or IV	NSCLC		199	T/T vs. G/G	1.05	0.66–1.67	0.91(log‐rank)	NS	19
Dong	2011	China	Asian	72%	Stage I‐IV	NSCLC		561	G/G vs. T/T + T/G	1.37	1.06–1.78	0.017	T/T + T/G risk	20
Ryan	2012	States	Caucasian and African‐American	92%	Stage I‐IV	NSCLC		197	T/T vs. G/G	0.8	0.51–1.24	0.31	NS	12
This study	2015	Japan	Asian	45%	Stage I	AC		179	T/G + G/G vs. T/T	2.23	1.07–4.65	0.033	T/T risk	

HR, hazard ratio; CI, confidence interval; Ref., Reference; NSCLC, non‐small‐cell lung cancer; NS, not significant; NA, not available; AC, adenocarcinoma; SQ, squamous cell carcinoma.

The T/T genotype was associated with poor survival in patients with aggressive bladder cancer 27, in line with our observations. The cited authors concluded that *p53* mutational status was of prognostic value, but, in this study, the *p53* mutation levels did not differ significantly by *MDM2* genotype (Table 3). Any prognostic utility of the SNP309 marker in gastric cancer, renal cell carcinoma, and breast cancer, remains controversial 28, 29, 30. Furthermore, of early‐stage cancers, only lung cancer has been analyzed 15, 17.

Regarding the *p53* Arg72Pro, it has been reported that the Arg/Arg variant encodes a highly proapoptotic protein, whereas the Pro/Pro variant has the opposite effect 31. We analyzed the associations between *p53* status (*p53* mutation and *p53* Arg72Pro) and c.309T>G of the *MDM2* gene, and consequently found that the T/T genotype was associated with worse OS among *p53* wild‐type group (HR = 3.69) and the *p53* Arg72Pro^31^ Arg/Arg + Arg/Pro group (HR = 2.99) (Fig. 3), although these results are underpowered due to small sample size. Our findings are consistent with those of Chien et al. about *p53* mutation status 17. *p53* function is considered normal (not inactivated) in patients with the T/T genotype, Arg/Arg + Arg/Pro, or *p53* wild‐type group compared to the 309G allele carrier, Pro/Pro, or *p53* mutant group. Furthermore, among patients in the abovementioned groups, the T/T genotype was associated with worse OS (HR = 4.39). These results suggest a positive interaction between the T/T genotype, *p53*Arg72Pro RR + RP, and *p53* wild type in increasing the risk of death.

The tumorigenic functions of *MDM2* in both p53‐dependent and ‐independent pathways are complicated, and analysis of one polymorphic variant may not address all the *MDM2* functions. The precise mechanism underlying the worse OS with the T/T genotype being associated with *p53* status remains unknown 32. However, tumors of *MDM2* T/T patients tended to be positive in LVI and pleural invasion (Table 3), which have been reported to be worse prognostic factors associated with tumor proliferation and aggressiveness 33, 34. These results indicate that the tumors of T/T patients in the stage I period might have overall malignant potential, although *p53* tumor suppressor function is normal. Based on our results, tumors that develop under normal *p53* might have a malignant potential rather than tumors that develop under abnormal *p53*, and genotyping of c.309T>G might simply be a selection tool for malignant potential for stage I lung AC.

Throughout this study, we found that c.309T>G was a predictive factor of postoperative survival among p‐stage I lung AC patients in a Japanese population. Analysis of *MDM2* polymorphism has several advantages over somatic cell mutations analysis. First, the *MDM2* 309T>G polymorphism can be used to predict which individuals are at an increased risk of death after surgery. Second, the assessment of an individual's polymorphism status does not require an extraction of tumor‐specific DNA. In this study, the *EGFR*,* KRAS*, and *p53* mutations were not independently associated with prognosis as previously reported 35. Although our findings need to be validated in prospective studies, c.309T>G would be a useful prognostic marker that is detectable at any stage of diagnosis or treatment and influences the therapeutic strategies. Furthermore, we had already established the Duplex SmartAmp method^22^ to detect c.309T>G with a single drop (5 *μ*L) of blood within 40 min from sample collection. If we can make this method more practical, we will detect this SNP more easily and quickly in any clinical situation.

This study possesses several limitations. First, we could gather data on OS but not on cancer‐specific survival because the sources of survival data did not indicate the cause of death, although it would be useful to know the cause of death especially for early‐stage cancer patients. Another limitation of our study is its retrospective nature, although blood sample collection was performed preoperatively, and the database was run prospectively. Therefore, patient populations might be biased. Finally, the sample size and number of events in this study might be too small to draw meaningful conclusions associated with *p53* status. Further prospective studies with a larger, more homogeneous study population would be desirable to abrogate these limitations.

In conclusion, to the best of our knowledge, this is the first study to analyze the effects of c.309T>G in the *MDM2* gene together with *p53* Arg72Pro as well as mutations in the *EGFR*. *KRAS* and *p53* genes on the prognoses of lung cancer patients. We conclude that the T/T genotype of c.309T>G affects OS in surgically resected stage I lung AC patients and represents an independent prognostic factor in a Japanese population. Further studies are warranted to clarify the biological importance of these findings and the usefulness of the *MDM2* 309T>G polymorphism as a predictive marker for therapy selection and outcome prediction in NSCLC.

## Conflicts of Interest

None declared.
